# Investigating the contribution of ventral-lexical and dorsal-sublexical pathways during reading in bilinguals

**DOI:** 10.3389/fnhum.2014.00507

**Published:** 2014-07-14

**Authors:** Reyhaneh Bakhtiari, Carol Boliek, Jacqueline Cummine

**Affiliations:** Department of Communication Sciences and Disorders, Faculty of Rehabilitation Medicine, University of AlbertaEdmonton, AB, Canada

**Keywords:** bilingualism, DTI, tractography, reading, ventral lexical, dorsal sublexical

## Abstract

Several studies suggest the existence of ventral-lexical and dorsal-sublexical systems for reading. The relative contribution of these pathways can be manipulated by stimulus type and task demands. However, little is known about how bilinguals use these systems to read in their second language. In this study diffusion tensor imaging (DTI) was used to investigate the relationship between white matter (WM) integrity and reaction time in a group of 12 Chinese–English bilingual and 11 age-matched English monolingual adults. Considering a dual-route model of reading, the following four tracts were isolated in both the left and right hemispheres using a tractography measurement approach. Ventral tracts included the uncinate fasciculus (UF) and the inferior longitudinal fasciculus (ILF). The dorsal tracts of interest were the arcuate fasciculus (AF) and the superior longitudinal fasciculus (SLF). A significant correlation between the reaction time in a reading task and the mean diffusivity (MD) value was observed in the right UF in both bilingual and monolingual groups. Moreover, in the bilingual group we observed significantly more positive relationships between reaction time and MD in the right AF, and bilaterally in the SLF. We concluded that the relative contribution of the dorsal system for reading is greater in bilinguals than monolinguals. Further, these findings implicate a role of the right hemisphere in reading.

## Introduction

From e-mails to text messages, written communication holds an increasingly important role in today's society. Given this increased reliance, one can imagine the devastating impact of reading impairments in daily communication. Thus far, much work has been done on the functional framework of reading (Pugh et al., 2000, 2001; Mechelli et al., 2005; Borowsky et al., 2006, 2007; Cohen et al., 2008; Binder et al., 2009; Levy et al., 2009; Price, 2010). Globalization raises the need to learn more than one language more than ever. However, little information exists on the underlying neural structural correlates of reading in bilinguals. This study sought to provide heuristically important information about underlying white matter (WM) integrity and its relationship to basic reading processes in adult bilinguals. Such knowledge adds to our understanding of both reading and reading impairments in bilingual individuals.

Research investigating brain plasticity through WM and gray matter (GM) in bilingual brains has been mixed. For example, some have reported increases in the density of GM in the left inferior parietal cortex (Mechelli et al., 2004), and the left putamen (Abutalebi et al., 2013), as well as increased GM volume in the left caudate (Zou et al., 2012) of adult bilinguals when compared to monolinguals. In contrast, Gold et al. (2013) did not observe any difference in GM volume between older or younger monolinguals and bilinguals, and similarly, Mechelli et al. (2004) found no differences in WM density between monolingual and bilinguals. Diffusion tensor imaging (DTI), is another method for studying WM *in vivo* and is based on local microstructural characteristics of water diffusion (Basser and Pierpaoli, 1996; Le Bihan et al., 2001; Basser and Jones, 2002). Fractional anisotropy (FA) is a quantitative index to represent direction-dependent diffusivity of water molecules (Basser and Pierpaoli, 2011). Several developmental and pathological factors such as axon myelination, diameter distribution, axon density, and architecture of WM fibers have an effect on this parameter. FA is a normalized value between zero and one, where higher FA values show higher diffusion directionality and therefore higher WM integrity (Pierpaoli et al., 2001; Beaulieu, 2002). Mean diffusivity (MD) is another measure that is the average of the eigenvalues of the diffusion tensor and reflects the magnitude of diffusion (Ben-Shachar et al., 2007). Myelination, volume of extracellular space, and fiber packing effect the value of MD (Norris, 2001; Beaulieu, 2002; Song et al., 2005; Scantlebury et al., 2014). Correlations between FA and MD values and behavioral performance can provide insight about the role of the underlying WM tracts and the corresponding cognitive tasks or abilities.

Using a DTI tractography approach, more WM integrity as reflected in increased FA values is observed in the left inferior fronto-occipital fasciculus (IFOF) of 8–11 years old early bilinguals (exposed to a second language from the birth) but not late bilinguals compared with monolinguals (Mohades et al., 2012). The authors proposed the acquisition age of the second language affects subsequent changes in the brain structure, because the main process of myelination in the human brain occurs in early childhood, before the second year of life. Using a Tract Based Spatial Statistics (TBSS) approach (Smith et al., 2006), Luk et al. (2011) observed higher FA values in the corpus callosum, which extended to the superior longitudinal fasciculus (SLF) and inferior longitudinal fasciculus (ILF) for late bilinguals 70 years of age. In early bilinguals (20–40 years old) García-Pentón et al. (2014) found more WM connectivity in two sub-networks in (i) the left frontal and parietal/temporal regions, and (ii) the left occipital and parietal/temporal regions and also the right superior frontal gyrus. On the other hand, reduced WM integrity (reduced FA values) also was shown for 65 year old late bilinguals bilaterally in the ILF, IFOF, the fornix, and multiple portions of the corpus callosum (Gold et al., 2013). Considering similar functional performance in both monolingual and bilingual groups, the authors proposed that bilingualism may contribute to cognitive reserves that mitigate effects of WM integrity declination at these population ages. Similarly, Cummine and Boliek (2013) observed regions of lower FA in late, 20–40 years old bilinguals, in the right IFOF and bilaterally in the anterior thalamic radiation (ATR). These authors also showed a FA-response time (RT) correlation in the caudate, which is consistent with the notion that bilingual individuals exercise more cognitive control when speaking in their second language (Zou et al., 2012). Notably, these authors also considered MD and reported increased MD for bilinguals in the right ATR and ILF when compared to monolinguals. Although it has been reported that MD varies little across brain tissue (Ben-Shachar et al., 2007), this measure was clearly able to detect microstructural differences between monolingual and bilingual individuals.

The research reviewed above describes the differences in WM between monolingual and bilingual individuals. We have little information about the relationships between cognitive performance and WM in bilingual individuals, and more importantly, how these relationships compare to monolingual counterparts. In this paper, we focus on the relationship between reading performance and underlying WM pathways. Several functional and structural studies on language research supports the existence and distinction between a ventral-lexical-sound-to-meaning pathway extending from occipital-temporal-frontal regions, and a dorsal-sublexical-sound-to-articulation pathway extending from occipital-parietal-frontal regions (Pugh et al., 2000; Jobard et al., 2003; Borowsky et al., 2006, 2007; Steinbrink et al., 2008; Friederici, 2009; Price, 2010). The ventral stream is purported to be a lexically driven path characterized by whole-word memory recognition for familiar words and exception words (e.g., letter strings with an atypical spelling-to-sound correspondence, *pint*). The dorsal stream is characterized as a sublexical pathway that integrates orthographic information (graphemes) with their phonological, morphological, and lexical-semantic counterparts. Hence, the dorsal route plays an important role when learning how to read by mapping letter-to-sound correspondence and less familiar regular words (e.g., words with a typical letter-to-sound correspondence, *munch*). Recent DTI work provides evidence that there are separate ventral and dorsal pathways important for lexical and sublexical processes, respectively (Saur et al., 2008, 2010; Friederici, 2009; Agosta et al., 2010; Lopez-Barroso et al., 2011). Dorsal pathways connect regions in the temporal lobe to the inferior frontal gyri by way of the arcuate fasciculus (AF), and SLF. The integrity of these tracts has been linked to rehearsal of phonological working memory (Lopez-Barroso et al., 2011), repetition-based tasks (Saur et al., 2008), and syntactic processing (Wilson et al., 2011), which is consistent with the notion of a dorsal-sublexical-sound-to-articulation pathway. The ventral pathway connects regions in the middle temporal gyri to the inferior frontal gyri by way of the extreme capsule, uncinate fasciculus (UF), and ILF. The integrity of these tracts has been linked to performance on comprehension based tasks (Saur et al., 2008) and reading (i.e., RT) of exception words (Cummine et al., 2013), providing further evidence for a ventral-lexical-sound-to-meaning pathway. Overall, the dual-pathway model provides a framework from which we can begin to explore the relationships between behavioral reading performance and underlying WM pathways in monolingual and bilingual adults.

As far as we know the relation between WM integrity and reaction times taken during a reading task has been only studied by Cummine and Boliek (2013) in bilinguals. The results of this work were an important step toward clarifying structural differences between monolingual and bilingual adults and providing preliminary information on brain-behavior relationships for different word types (exception words vs. regular words); hence the exploratory voxel-wise approach was used to address both research questions in that paper. However, we still do not know to what extent the underlying WM pathways are related to ventral-lexical and dorsal-sub-lexical pathways in monolingual and bilingual adults, nor do we know how these relationships differ as a function of language status. The present study was designed to advance our understanding of the underlying neural mechanisms associated with reading in bilinguals and monolinguals, within the context of the dual-pathway model of reading. In this study we investigate the relationship between WM integrity of ventral and dorsal pathways and task performance during reading lists of words with different proportions of regular words (stimuli that can utilize both ventral-lexical and dorsal-sub-lexical pathways) and exception words (stimuli that must utilize the ventral-lexical pathways).

## Materials and methods

### Participants

Eleven right-handed, English first language speakers (mean age ± SD: 28.5 ± 8.8 years, range: 21–50, 6 female, 5 male)^1^, and 12 bilingual Chinese–English speakers (24.25 ± 4.1 years; range: 19–33, 8 female, 4 male) participated in this study ^2^. The groups did not differ on age [*t*_(21)_ = 1.48, *p* = 0.152]. Bilingual speakers were considered late bilinguals because they received written English instruction after 5 years of age. All participants were right-handed with normal or corrected to normal vision. Consent was obtained according to the Declaration of Helsinki (2013, http://www.wma.net/en/10home/index.html) and the experiment was performed in compliance with the relevant laws and institutional guidelines and was approved by the University of Alberta Health Research Ethics Board.

### Behavioral data

Behavioral data were collected in a normally lit room, where stimuli were presented on a computer monitor using EPrime software (Psychology Software Tools, Inc., http://www.pstnet.com). Each participant read three lists of words containing both exception words and regular words. Each trial began with a 100 ms fixation cross, followed by a word. Words in each list were randomly presented. Participants were instructed to name each stimulus aloud as quickly and accurately as possible, and they were given 1800 ms to name the stimuli.

Our rationale for manipulating the contribution of the dorsal system via familiar regular word stimuli was two-fold. First, our previous work that examined the relationship between reading performance and WM pathways (in monolinguals), reported very weak relationships between sub-lexical-dorsal tracts and nonwords/pseudohomophones (stimuli typically used to measure sub-lexical processing) and stronger relationships between ventral WM tracts and familiar stimuli (exception words and regular words; Cummine et al., 2013). Second, using familiar stimuli to explore the relationships between ventral-lexical and dorsal-sub-lexical pathways is arguably more ecologically valid in skilled adult readers. The manipulation of list proportion served to impact the relative contribution of lexical and sub-lexical processing under familiar reading. The conventional approach (i.e., correlating mean RT from pure lists of stimuli with brain measures) has been reported previously in the literature (at least for monolinguals; Cummine et al., 2013). While this approach is useful for studying specific processes in isolation (i.e., exception words as a “pure” measure of lexical processing) it is an extreme reading condition that (1) does not reflect natural reading conditions and (2) is limited in its ability to shed light on the dynamic contribution of the lexical and sub-lexical systems. We hypothesized that relationships between FA/MD of lexical-ventral tracts and behavioral RT would be evident in all word-type proportion conditions, given that the lexical-ventral stream accurately processes familiar letter strings. In a condition where participants are naming a list of words that consists of 25% exception words and 75% regular words (hereafter referred to as 25EXC:75REG), information from the dorsal-sub-lexical system would be accurate 75% of the time (i.e., in the case of the regular words), and thus, we hypothesize that we will also see a relationship between behavioral RTs and FA/MD of dorsal tracts. Given that information from the sub-lexical-dorsal stream would be inaccurate 50 and 75% of the time in the a reading condition that had 50% exception words and 50% regular words (hereafter referred to as 50EXC:50REG) and 75% exception words and 25% regular words (hereafter referred to as 75EXC:25REG), respectively, we hypothesized that the relationship between behavioral RT and FA/MD of dorsal tracts would be negligible in the 50EXC:50REG and 75EXC:25REG conditions.

The proportions of exception words and regular words in a list were manipulated across three conditions: 25EXC:75REG had 25% exception words and 75% regular words, 50EXC:50REG had 50% exception words and 50% regular words, and 75EXC:25REG had 75% exception words and 25% regular words. Each list had 60 stimuli, and each stimulus appeared only once within the whole experiment. The stimuli in each list were monosyllabic and between 3 and 7 letters in length. Kucera-Francis (KF) mean frequency for each list ranged between 99 and 200. The exception and regular words in each of the word lists were matched for length, KF frequency, and onset phoneme. The characteristics of the 25EXC:75REG list were as follows: exception word length ranged from 3 to 6 (Mean = 4.47); regular word length ranged from 3 to 6 (Mean = 4.33), exception word frequency ranged from 2 to 536 (Mean = 140.33); regular word frequency ranged from 1 to 1044 (Mean = 175.47). The characteristics of the 50EXC:50REG list were as follows: exception word length ranged from 4 to 6 (Mean = 4.47); regular word length ranged from 4 to 6 (Mean = 4.43), exception word frequency ranged from 1 to 2764 (Mean = 163); regular word frequency ranged from 1 to 2988 (Mean = 194). The characteristics of the 75EXC:25REG list were as follows: exception word length ranged from 4 to 6 (Mean = 4.56); regular word length ranged from 4 to 6 (Mean = 4.8), exception word frequency ranged from 2 to 1599 (Mean = 144.47); regular word frequency ranged from 4 to 1599 (Mean = 279.33). The lists were presented in one of two orders, either “75EXC:25REG-50EXC:50REG-25EXC:75REG” or “25EXC:75REG-50EXC:50REG-75EXC:25REG.” The 50EXC:50REG condition was always presented in the middle of the sequence as it was akin to a “neutral” condition. List presentation was alternated for each participant. RT measured by voice onset and accuracy (ACC) were recorded for each participant while reading each list.

### DTI data acquisition

Data were acquired using a 1.5 T Siemens Sonata MRI scanner. Head stabilization was achieved by foam padding, and all participants wore earplugs to attenuate noise. Diffusion-weighted data with 13 directions (12 diffusion-weighted+1 T2) were acquired using a dual spin-echo single-shot echo-planar imaging sequence with the following parameters: *TR*/*TE* = 4600/96 ms, *b* = 1000 s/mm^2^. Twenty-seven 4-mm thick axial slices were obtained with FOV of 256 × 256 mm^2^, an acquisition matrix 128 × 128, which results in, 2 × 2 mm^2^ of in-plane image resolution, and, with 75% phase partial Fourier zero-filled.

### Tractography

Diffusion tensor tractography is a method of identifying and visualizing a continuous three-dimensional trajectory by sequentially piecing together the estimates of fiber orientation from the directionality of individual voxels (Conturo et al., 1999; Mori et al., 1999; Basser et al., 2000). Using this method, equivalent fiber connections across individuals can be compared even though the precise location of the tract varies (Wakana et al., 2004; Mukherjee et al., 2008).

**Figure 1:**
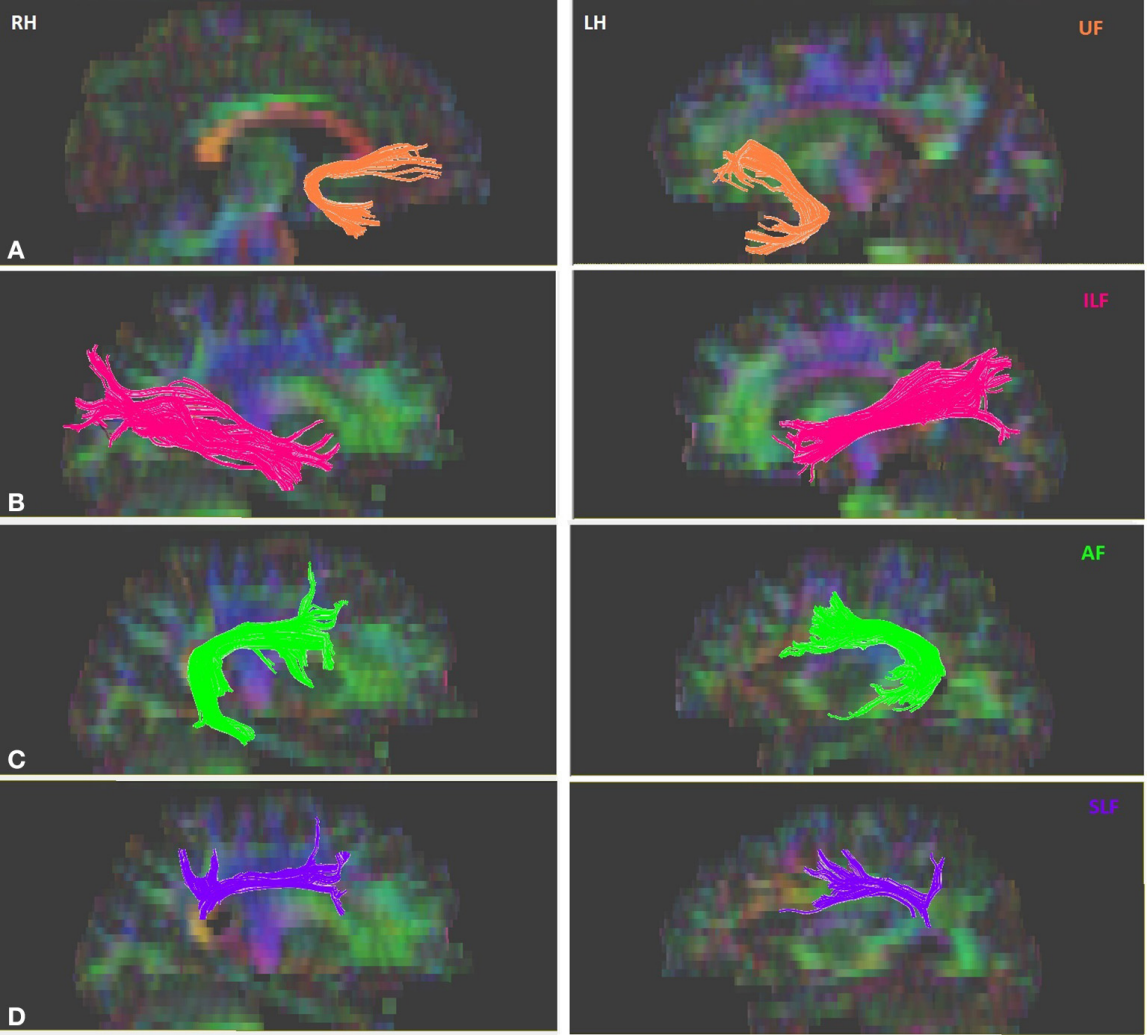
**Sample of isolated tracts in the left (LH) and right (RH) hemispheres. (A)** Uncinate fasciculus (UF), **(B)** Inferior longitudinal fasciculus (ILF), **(C)** Arcuate fasciculus (AF), **(D)** Superior longitudinal fasciculus (SLF).

Tractography for the tracts of interest was performed for each participant on DTIstudio, using a multiple region of interest (ROI) approach and referencing the protocols (Wakana et al., 2007; Zisner and Phillips, 2009a,b,c). The multiple ROI approach utilizes existing anatomical knowledge of tract trajectories in order to reconstruct said tracts. Fibers that penetrate the manually defined ROIs are assigned to the specific tracts associated with those ROIs (Wakana et al., 2007; Agosta et al., 2010). First, DTI images were co-registered to each other and the b0 image. Given that ROIs were drawn and tractography was performed in native space, the DTI images were not spatially normalized. Further, the mean translation parameters were evaluated in each participant and the average translation values were found to be less than voxel size (mean ± std, [min, max]) (*x*: 0.005 ± 0.085, [−0.175, 0.296], *y*: 1.043 ± 0.275, [0.505, 1.664], *z*: 0.879 ± 0.109, [0.403, 1.390] mm). Given that these values were within the slice thickness of the images, no motion correction was applied in order to avoid introducing potential artifacts or loss of sensitivity. The primary ventral tracts of interest were the left and right ILF, and UF. The primary dorsal tracts of interest were the left and right AF and SLF. Continuous Tracking (FACT) algorithm using principle vector and FA map was used for tract construction. An FA threshold of 0.25 was used to initiate and continue tracking; the maximum angle threshold was 70°. With reference to the protocol outlined in Wakana et al. (2007), three types of ROI operations within DTIstudio were used for fiber tracking: “OR,” “AND,” and “NOT.” The “OR” function selects all the fibers that penetrates a particular ROI, the “AND” function selects only those fibers that penetrate the defined ROIs, and the “NOT” function removes any fibers that penetrate the defined ROI. A sample figure for each of the isolated tracts is shown in Figure 1. The left UF in one of the subjects, the right UF and the right AF in another subject could not be isolated. After the reconstruction of the tracts, DTIstudio software computed the mean FA and three eigenvalues that were used to calculate MD values, of each tract for each individual, which were then correlated with behavioral measures.

### Reliability of tractography analysis

Six participants were randomly selected from the pool of participants and DTI tractography was performed a second time to check the intra-rater reliability on the following tracts: left/right UF, and left/right SLF. Overall, it was found that the FA value obtained from the second tractography analysis correlated strongly with the FA value from the first tractography analysis, *r*_(5)_ = 0.95, *p* < 0.002.

## Results

### Behavioral analysis

Average correct reading RTs and accuracy for both monolinguals and bilinguals for reading each list are displayed in Table 1. Independent-samples two-tailed *t*-tests were used to compare between-group differences. To correct for multiple comparisons, only *p*-values that survived the False Discovery Rate (FDR) control are deemed significant. The appropriate *p*-value is reported base on the result of Levene's test for equality of variances. There were no differences in reaction time for reading different lists between monolingual and bilingual groups (Table 1). Accuracy of bilingual participants was significantly less in all three reading lists compared with the monolingual group (*p* < 0.05). In both groups RT for reading the 25EXC:75REG list was the fastest, whereas it was the slowest for reading the 50EXC:50REG list. In the monolingual group, the RT for the 25EXC:75REG condition was significantly faster than RT in the 50EXC:50REG condition [*t*_(10)_ = 3.8, *p* = 0.003]. In the bilingual group, RT for the 25EXC:75REG list was significantly faster than RT for both the 75EXC:25REG [*t*_(11)_ = 3.0, *p* = 0.012] and the 50EXC:50REG [*t*_(11)_ = 5.5, *p* < 0.001] lists.

**Table 1:** **Mean ± Std of average reaction time (RT) and accuracy of monolinguals and bilingual groups for reading the three lists**.

	**Average RT (ms)**			**Accuracy**		
	**Monolinguals**	**Bilinguals**	***p*-value**	**Monolinguals**	**Bilinguals**	***p*-value**
75EXC:25REG	537.5 ± 87.0	599.3 ± 114.0	0.152	0.97 ± 0.02	0.93 ± 0.06	0.047
50EXC:50REG	545.8 ± 57.7	603.7 ± 112.9	0.161	0.96 ± 0.02	0.87 ± 0.12	0.020
25EXC:75REG	514.5 ± 60.2	566.7 ± 119.0	0.143	0.97 ± 0.03	0.91 ± 0.04	0.001

### Tractography analysis

#### Relationships between FA and MD in monolingual and bilingual groups

One-tailed Pearson's correlations were conducted to find linear relationships between FA, and MD, values along the tracts in lexical-ventral and sub-lexical-dorsal pathways (UF, ILF, AF, and SLF) and RT when reading the three lists. Correlational analyses found no significant relationship between mean FA obtained from the tracts of interest and reading conditions for either the bilingual or monolingual groups. The correlations between MD values in the mentioned tracts and RT are given in Table 2. FDR control was used to correct for multiple comparisons. To make sure, that outliers did not affect our data, “leave one out” tests were performed on all correlation cases. The correlation values that remained significant after FDR correction of each condition and 90% of “leave one out” tests are reported and denoted by ^*^ in Table 2.

**Table 2:**
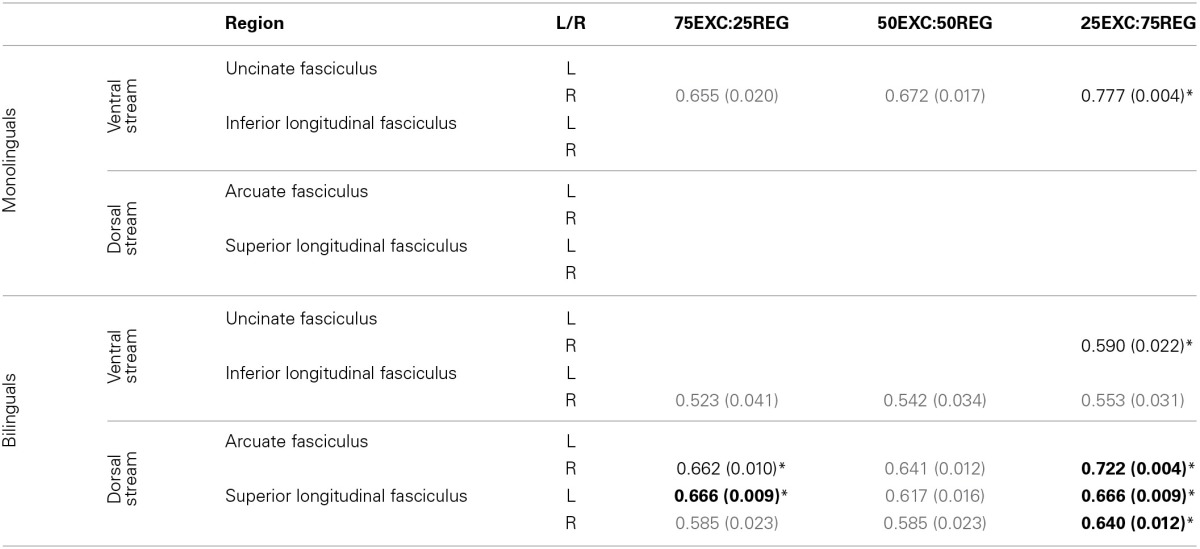
**Pearson's *r*-value (*p*-value) for correlation between MD values and RT in monolingual and bilingual groups**.

The correlations survived after FDR correction and 90% of leave one-out tests are denoted by ^*^, vs. those who did not (gray color). The values in bolded face show significantly higher correlation in the bilingual group than in monolingual group.

***Monolinguals***. A strong positive correlation was observed between MD values in the right UF and RT for reading 25EXC:75REG list [Pearson's *r*_(9)_ = 0.777, *p* = 0.004]. There was also a trend for a positive correlation between MD in the right UF with RT in 50EXC:50REG, and 75EXC:25REG conditions.

***Bilinguals***. The MD values were positively correlated with 25EXC:75REG RT in the right UF [Pearson's *r*_(11)_ = 0.590, *p* = 0.022], right AF [Pearson's *r*_(11)_ = 0.722, *p* = 0.004] and bilaterally in the SLF [left SLF: Pearson's *r*_(11)_ = 0.666, *p* = 0.009, right SLF: Pearson's *r*_(11)_ = 0.640, *p* = 0.012] (Table 2). A trend toward a positive correlation between MD values in the right ILF, right AF, and bilaterally in the SLF was observed for RT of the 75EXC:25REG and 50EXC:50REG lists, and between MD values in the right ILF and RT of the 25EXC:75REG list.

#### Group comparisons

**Figure 2:**
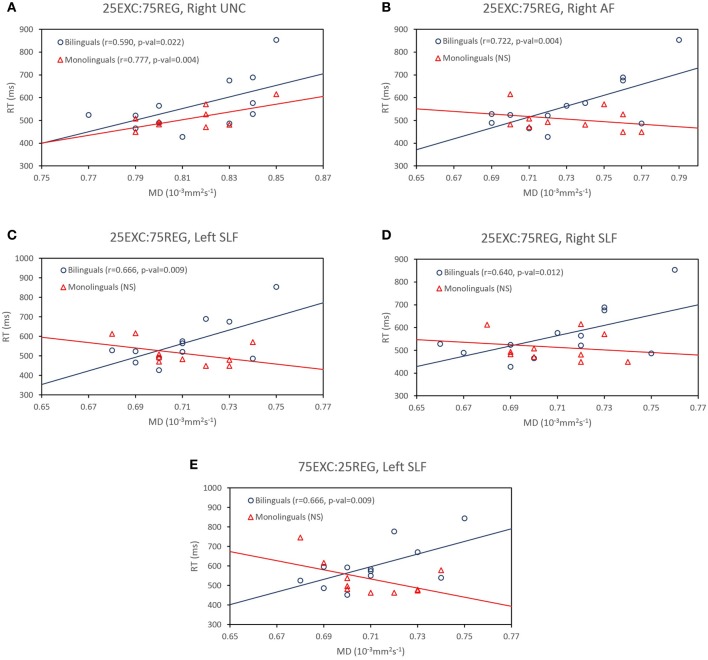
**(A)** Significant correlations between MD values in the right uncinated fasciculus (UF) and reaction time in the 25EXC:75REG list in both monolingual and bilingual groups. **(B–D)** Significantly higher correlations in bilinguals than in monolinguals between reaction time in the 25EXC:75REG list and MD values in the right arcuate fasciculus (AF), right left and superior longitudinal fasciculus (SLF), respectively, **(E)** Significantly higher correlations in bilinguals than in monolinguals between reaction time in the 75EXC:25REG list and MD values in the left superior longitudinal fasciculus (SLF)

Follow-up tests were conducted to test whether reported correlations in the monolingual and bilingual groups were significantly different from each other. As such, 50,000 Monte Carlo permutation tests (to reduce the margin-of-error to below 4% of the nominal alpha) were conducted to test whether the correlation between MD and RT was significantly different between monolingual and bilingual participants (http://fsl.fmrib.ox.ac.uk/fsl/fslwiki/Randomise/Theory). The correlation values that were significantly higher in bilingual than in monolingual individuals are bolded in Table 2. Correlation values between MD values in the right AF, and bilaterally in the SLF and RT in 25EXC:75REG list, and MD values in the left SLF and RT in 75EXC:25REG list were significantly higher in bilingual groups than in monolingual groups. In Figure 2, scatter plots for MD values and RT in these tracts, along with the right UF, in which both group have high correlation between MD values and RT are shown.

## Discussion

In this study we provide evidence for the relationship between WM integrity (i.e., MD) and reading performance that varied as a function of language status and list composition (i.e., lexical/sub-lexical contribution). More specifically, we demonstrate that WM tracts underlying the ventral stream are important in lexical processing in both bilinguals and monolinguals; but dorsal-sub-lexical WM tracts are only related to reading performance in bilingual participants.

With respect to the behavioral data, the manipulation of proportions succeeded in modulating the contribution of lexical and sub-lexical information. More specifically, for both monolinguals and bilinguals, RTs in the 25EXC:75REG condition and 75EXC:25REG condition were relatively fast with high accuracy, indicating an appropriate contribution/modulation of lexical and sub-lexical systems was achieved (see Monsell et al., 1992, for another demonstration of lexical/sub-lexical modulation as a function of list type). Interestingly and somewhat unexpectedly, the 50EXC:50REG condition resulted in the slowest RTs. Based on previous research that has evaluated list composition effects (Monsell et al., 1992; Lupker et al., 1997), we hypothesize that this finding is a reflection of processing and evaluating information within both the ventral-lexical and dorsal-sub-lexical systems. This seems somewhat counterintuitive given that the dorsal-sub-lexical system provides an incorrect pronunciation 50% of the time (in the case of exception words). However, if we consider that regular words can utilize both lexical and sub-lexical processing, while exception words utilize primarily lexical processing, this discrepancy in the “best strategy” to implement (i.e., two systems vs. one system) may be driving the inability to “settle” on an efficient processing strategy. In this scenario, participants would need to continually monitor information to prevent an incorrect overt production. Although beyond the scope of the present paper, a breakdown of the RT and accuracy for each stimulus type in each condition would provide further verification of this conclusion and is an avenue for future research.

In both monolingual and bilingual groups we observed a positive correlation between MD values in the right UF and RT for the 25EXC:75REG list. The UF is a part of ventral-lexical stream, which connects the orbitofrontal region to anterior temporal cortex (Catani et al., 2002; Schmahmann et al., 2007). This region is involved in processing lexical stimuli, semantic associations, and aspects of reading (Grossman et al., 2004; Marchina et al., 2011). Considering that all regular and exception words used in the lists are familiar, in the 25EXC:75REG condition both the ventral-lexical and dorsal-sublexical streams are involved in word processing in a parallel fashion. As the conflict between outputs of these systems is minimized (25% of the cases), no higher level monitoring system is active to select the correct output, but instead reading proceeds in a more-or-less automatic fashion. Notably, we did not see this same UF MD-RT relationship in the other two reading conditions, which also contained highly familiar stimuli. One possible explanation for this result is that UF is sensitive to *automatic* and/or unconstrained lexical access. More specifically, in the 75EXC:25REG and 50EXC:50REG conditions, the conflict between the outputs of the lexical and sublexical systems is substantial (75 and 50% of the words, respectively), so the outputs from both systems should be carefully monitored, which adds processing time as reflected in the generally longer RTs for these two lists. Overall, our results are in line with previous studies that show that UF is important for processing familiar information (Papagno et al., 2011; Wilson et al., 2011; Cummine et al., 2013).

Here we showed in the bilingual group more positive correlations between RT and MD values in the right AF (dorsal-sublexical), and bilaterally in the SLF (dorsal-sublexical) in 25EXC:75REG condition, and in the left SLF and 75EXC:25REG condition, when compared to monolinguals. As there is no difference in RT between the two groups, these increased MD-RT relationships for bilinguals in dorsal streams may be reflective of more generalized mechanisms that support basic reading processes in the secondary language. The dorsal-sublexical system includes AF that connects caudal temporal cortex and inferior parietal cortex to locations in the frontal lobe and SLF, which connects frontal lobes to temporal and parietal lobes (Wakana et al., 2004). Phonological awareness has been shown to be correlated with higher WM integrity in the left AF in adults (Rolheiser et al., 2011; Vandermosten et al., 2012a) and children (Saygin et al., 2013) (but see Yeatman et al., 2011). Further, the left AF has been shown to be less integrated in individuals with dyslexia (Vandermosten et al., 2012b) and damage to the left SLF has been linked to a profound deficit in learning to read, whereas damage to the right SLF causes visuospatial deficits (Rauschecker et al., 2009). Together these studies emphasize the distributed network of brain regions and subsequent WM pathways that can contribute to reading skills. The distributed pattern of MD-RT relationships reported here suggests bilingual participants make use of this larger brain network when reading in their second language. Our results show that, for both groups, MD is a better predictor of reading RT. It could be conferred that factors affecting MD value such as membrane permeability, creation of intracellular, compartments and fiber re-organization, rather than WM structures, are more related to RT in the current reading context (Norris, 2001; Beaulieu, 2002; Song et al., 2005; Scantlebury et al., 2014). However, it should be noted that all diffusion measures are calculated from the same three eigen-vectors, and thus the values are not totally independent, and the exact relationship between WM structural features and these measures is not well understood.

Our “distributed network” finding also fits well with current fMRI literature on bilingualism that reports bilinguals have greater brain activity in the dorsal-sublexical regions during reading compared with monolinguals. For example, Kovelman et al. (2008a) found that Spanish–English bilinguals showed greater activity in the left inferior frontal cortex during sentence judgment task when compared with English monolinguals. Kovelman et al. (2008b) reported greater functional Near-Infrared Spectroscopy (fNIRS) activity bilaterally, and particularly in the right hemisphere in the dorsolateral prefrontal cortex (BA 46/9) and inferior frontal (BA 47/11), working memory/attention prefrontal cortex area during semantic judgment task. The same pattern of findings (i.e., increased activation in dorsal pathways) has been reported for Spanish–Catalan bilinguals (Rodriguez-Fornells et al., 2002), German–Spanish bilinguals (Rodriguez-Fornells et al., 2005), English–Latin based/Greek bilinguals (Jones et al., 2012), English–Chinese bilinguals (Tan et al., 2003). It has been argued that increased brain activation in bilingual participants, while reading in their second language, is attributed to the need of more monitoring control to avoid competition errors of two languages, and also increased task demand due to less rehearsal of each word (Jones et al., 2012). This may be one explanation for the greater number of relationships between WM integrity and behavioral RT performance in the bilinguals in the current study, compared to the monolinguals.

While the importance of the right UF in bilingual participants has been reported previously (Luk et al., 2011), the extensive right hemisphere MD-RT relationships warrant further discussion. With respect to the bilingual participants, the right hemisphere relationships might reflect skilled reading acquisition as it has been suggested that the involvement of the right hemisphere in reading and language tasks decreases as a function of skill acquisition (Raboyeau et al., 2004). While our groups were similar on RTs, the bilingual participants did show lower accuracy as compared to the monolingual participants, albeit with a similar pattern across the conditions, which may be a reflection of their reduced exposure to the language. As mentioned previously, the bilinguals in this study are classified as late-bilinguals, being exposed to English after the age of five. With respect to the monolingual participants, only the right UF showed an MD-RT relationship. We know from our previous work that pure lists of regular and exception words, which provide the most optimal scenario for participants to settle into an effective reading strategy, produce MD-RT relationships in the left UF (Cummine et al., 2013). We argued above that the MD-RT relationship in the right hemisphere for the 25EXC:75REG condition was a result of the automaticity of the condition. Specifically, discrepant pronunciations from the sub-lexical system would only occur 25% of the time (i.e., for the exception words). However, there is still *some* conflict, which may be why we do not see the left hemisphere relationship that was reported in Cummine et al. (2013). Given the well accepted premise that language is left hemisphere dominant, Cummine et al. (2013) did not evaluate right hemisphere tracts and thus it is unknown to what extent the “pure” list reading tasks are related to right hemisphere pathways as well. Ultimately, while diffusion imaging can provide some evidence for the underlying mechanisms involved in reading, detailed specification related to the reading pathway use and its modulation will be best clarified in combined fMRI-DTI studies. In sum, our data highlight the importance of evaluating both left and right hemisphere pathways in the study of structural plasticity as it relates to reading both in monolingual and bilingual populations.

## Conclusion

In this study we compared association between WM integrity and reading performance in adult Chinese-English bilinguals and English monolinguals. Direct group comparison revealed no group differences in reaction time between monolingual and bilingual groups. In both groups, there was a positive correlation between WM integrity in the right UF and reading speed, which suggests the importance of this ventral/lexical tract in reading. Interestingly, our results demonstrated that other dorsal/sublexical tracts (the right AF, and bilaterally in the SLF) were also important during reading in the bilingual group. These observations indicated that recruitment of widespread reading network in bilinguals may be the result of higher task demand for these participants.

### Conflict of interest statement

The authors declare that the research was conducted in the absence of any commercial or financial relationships that could be construed as a potential conflict of interest.

## Footnotes

^1^We tested all reported relationships in the manuscript with and without the participant who was 50 years old. The removal of this participant had no effect on between group differences on RT, accuracy, FA, MD, or correlations between RT and WM measures.

^2^A subset of data from these participants have been reported on in a previous paper using a different analysis technique to address the question of FA/MD differences between monolinguals and bilinguals (Cummine and Boliek, 2013).
